# Report on Otology

**Published:** 1871-12

**Authors:** Samuel J. Jones

**Affiliations:** Professor of Ophthalmology and Otology, Chicago Medical College


### REPORT ON OTOLOGY.

By SAMUEL J. JONES, A. M., M. D.,
Professor of Ophthalmology and Otology, Chicago Medical College.

[From Transactions Illinois State Medical Society.]

Your Committee having, in the last annual report, given a general view of Otology, confine the present one chiefly to matters of material interest pertaining to the progress made, more especially during the last year, regarding diagnosis and the treatment of some of the more frequent diseases of the ear.

One of the most evident assurances of advancement, in diagnosis, is the gradual diminution in the number of cases formerly classified as nervous deafness. The cases in which we must regard the primary lesion as of nervous origin are but few.

The usefulness of the ordinary tuning-fork of musicians is becoming more extended as an aid in diagnosis. Next to inflating the Eustachian tubes, it is, perhaps, the most convenient mode of determining whether or not the Eustachian tubes be closed. If such closure exists, pressing the tragus back, so as to close the meatus, will render the sound produced by placing a vibrating tuning-fork on the patient's forehead less distinct, whilst, if the tube be open, such pressure on the tragus will increase considerably the sound heard. If, by inflating the tube, air enters freely, and yet pressure as described (on the tragus) does not make the sound louder, thc prognosis is less favorable than where it increases it.

In the last report, special consideration was given to "chronic catarrh of the middle ear," in the belief that its frequency in this region of country entitled it to such prominence. Another year's experience in the treatment of that class of cases, seems to add confirmation to the view then expressed, and in the treatment of them progress is still being made. For much of this we are indebted to Hinton, of London, who, with a view to rid the middle ear of altered secretions there accumulated, incises the membrana tympani and washes that cavity through the external meatus, passing a stream down through the Eustachian tube and out through the nares, by inclining the head of the patient forward, and having him keep his mouth open (as in using the nasal douche) when the soft palate is closed up against the pharynx.

The method of ridding the middle ear of its accumulation, by incising the membrana tympani, and thus syringing it, was practiced several years ago, but the stream was allowed to pass into the throat, making the process so disagreeable to the patient as to have aided in bringing the operation into disuse. Hinton seems to have succeeded, in a very simple way, in removing one of its most disagreeable features. His process is the reverse of that of Gruber, of Vienna, who passes the stream in through the nostril and out through the external meatus. Marked improvement follows the operation in many of these cases.

The apparatuses designed for making applications of fluids or medicated air to the affected surface of the middle ear, have been considerably simplified, and the means of ascertaining definitely of their passing into that cavity better established.

The importance of having the Eustachian tube in such condition that air can enter it with each repiratory movement, is impressing itself more fully, and renders advisable the frequent using of the Valsalvian method of inflating in all cases of thickening of the mucous membrane of the tube.

Tröltsch dwells with considerable emphasis on a class of cases which he calls, "Purulent Aural Catarrh in Children," and which seems to have escaped general attention. Most that is known regarding them has been obtained by *post mortem* examination by Tröltsch, Wreden, and a few others. The large proportion of cases in which pus has been found in the cavity of the tympanum, and in the mastoid cells, when not suspected during life, seems to merit more consideration of them than has heretofore been given them, and may explain many of those head symptoms children are so frequently affected with, and which are often attributed to vague supposable causes. As kindred to these Tröltsch speaks of another class, which he designates "Infantile Otitis," but which, in many respects, especially in its earlier stages, is identical with the aural catarrh before referred to, and only when the inflammation has become more diffuse deserves a different designation.

These cases naturally lead us to consideration of the conclusions he has drawn, that, in many instances, those mysterious cases which happen to all physicians who treat diseases of children and in which a satisfactory diagnosis is not arrived at before death, but where the convenient cause of "teething" is made to account for a train of symptoms that precede death, are ascribable to disease originating in the ear and secondarily affecting the brain. In determining this point exclusion serves us well. Evidence of great pain is manifested in the continuous crying of the child, who often is unable to speak, and can give no idea of its location. That it is not located in the alimentary canal, is determined by the absence of the physicial signs of inflammation there; that it is not yet seated in the brain, may be likewise determined, and thus, when once attention is drawn to the probable locality of the lesion, and it is remembered how large a proportion of children evince this condition after death, careful examination will render diagnosis of such cases easier.

In the treatment of discharge of pus from the ear, whether from the external meatus or from the middle ear through a perforated membrana tympani, syringing the ear thoroughly with a solution of sulphate of zinc, with the addition of some carbolic acid, gives, perhaps, more satisfactory results than any other remedial measure. Especially is the carbolic acid serviceable in cases where the parasite asperquillus glaucus is present. Regarding this, Schwartze says, "it appears probable that this vegeteble parasite is more frequently a cause of the obstinate, frequently relapsing, and chronic inflammation of the external auditory canal than is generally supposed." Investigation of this subject, by Green, of Boston, and others, has led to similar conclusions.

The use of electricity in the treatment of aural disease, is both commended and condemned by aural surgeons. The truth in this case, as in many others, probably lies in a mean between either extreme view. The chief objection to its use lying in the fact that we have not yet learned to discriminate sufficiently accurately as to the conditions of its applicability, and, instead of condemning it, further investigation as to the laws of its action on the human system is desirable. Some curious phenomena evinced themselves in experimenting upon the effects of electricity upon the auditory apparatus and have led to the inquiry whether or not there exists "accommodation" in the ear, as we have it in the eye, whether some muscular action in the ear is not exerted in a similar manner yet to be investigated.

Reasoning from the effects of strychnia upon the nervous system generally, it naturally would be suspected that it might be employed with advantage in those cases where the function of the auditory nerve is believed to be impaired, but experience seems to show that the ordinary effects of this drug are not exerted on nerves of special sensation. Hence the disappointment that has resulted in the cases where employed to restore the action of the auditory nerve, as well as in impairment of the function of the optic nerve.

The introduction of chloracetic acid as a caustic, has given us another convenient means of removing aural polypi; being readily soluble in water, it may, after having remained sufficiently long in contact with the polypus, be readily washed out of the meatus by syringing, without serious effect on the surrounding tissue.

Your Committee would gladly dwell more at length on diseases of the ear did the time of the Society admit. Their great frequency and importance merit such consideration.

When such authority as Tröltsch is led, by his experience, to the belief that there are more ear cases than eye cases, and to assert, as his conviction, that not more than one in every three persons, between the ages of twenty and forty years, possesses strictly normal hearing in both ears, we must feel convinced, that if the number be but one-half as great as represented by him, their frequency entitles them to more study than is accorded them by the general profession. As to their importance, we readily recognize the great disadvantage that persons labor under whose faculty of hearing has become greatly impared, and to what extent they are disqualified for many of the vocations of life. The earnestness with which these sufferers plead for relief shows how deeply they feel their loss.

It may not be amiss to conclude this report with some general suggestions as to the proper mode of conducting examinations of the ear.

Of first importance, is careful inspection of the membrana tympani. The simple means of illumination now at our command gives us all needed facility for that purpose. We must familiarize ourselves with the normal appearance of the membrane, during life, by frequent inspection of it in health, that we may detect variations from that standard in disease; then, having recognized these changes, we must learn to interpret their meaning. As well might an effort be made to study the appearance of a healthy cornea on the dead subject, as to try to get a correct idea of the appearance of the healthy membrane of the drum by inspecting it after the changes in it which occur after death. We must regard the membrane of the drum as giving us the key to diagnosis of disease of the ear, and further investigation as but supplementing such inspection of it. An elastic tube placed with one end in the ear of the surgeon, the other being in the ear of the patient, will act as the stethoscope does in examination of the chest, and convey to the ear of the surgeon the sound produced by air passing through the Eustachian tube into the middle ear, that sound being influenced by the condition of the parts through which the air passes, and a practiced ear of the surgeon will enable him to interpret the characteristic of the sound, as readily as may be done in auscultation of the chest.

